# Microbial Contribution to Wine Aroma and Its Intended Use for Wine Quality Improvement

**DOI:** 10.3390/molecules22020189

**Published:** 2017-01-24

**Authors:** Ignacio Belda, Javier Ruiz, Adelaida Esteban-Fernández, Eva Navascués, Domingo Marquina, Antonio Santos, M. Victoria Moreno-Arribas

**Affiliations:** 1 Department of Microbiology, Biology Faculty, Complutense University of Madrid, 28040 Madrid, Spain; ignaciobelda@ucm.es (I.B.); javiru02@ucm.es (J.R.); dommarq@ucm.es (D.M.); 2 CIAL-Institute of Food Science Research (CSIC-UAM), Dpt. Food Biotechnology and Microbiology, 28049 Madrid, Spain; adelaida.e.fernandez@csic.es; 3 Department of Food Technology, Escuela Técnica Superior de Ingenieros Agrónomos, Polytechnic University of Madrid, 28040 Madrid, Spain; enavascues@agrovin.com

**Keywords:** wine aroma, wine flavour, wine microbiome, yeast, non-*Saccharomyces*, lactic acid bacteria, oral microbiota, genetic and transcriptional directions

## Abstract

Wine is a complex matrix that includes components with different chemical natures, the volatile compounds being responsible for wine aroma quality. The microbial ecosystem of grapes and wine, including *Saccharomyces* and non-*Saccharomyces* yeasts, as well as lactic acid bacteria, is considered by winemakers and oenologists as a decisive factor influencing wine aroma and consumer’s preferences. The challenges and opportunities emanating from the contribution of wine microbiome to the production of high quality wines are astounding. This review focuses on the current knowledge about the impact of microorganisms in wine aroma and flavour, and the biochemical reactions and pathways in which they participate, therefore contributing to both the quality and acceptability of wine. In this context, an overview of genetic and transcriptional studies to explain and interpret these effects is included, and new directions are proposed. It also considers the contribution of human oral microbiota to wine aroma conversion and perception during wine consumption. The potential use of wine yeasts and lactic acid bacteria as biological tools to enhance wine quality and the advent of promising advice allowed by pioneering -omics technologies on wine research are also discussed.

## 1. Introduction

Wine is a special complex mix of chemistry, biology and culture where microorganisms play a critical role. The chemistry of grape and wine flavour has been widely studied due to the complexity of the volatile compounds contributing to wine aroma. Wine flavour comprises taste, aroma and visual attributes, aroma (smell) being the major contributor to the overall flavour perception [1]. Wine aroma compounds can be divided, depending on their origin, into: grape (varietal aromas), alcoholic and malolactic fermentation (fermentative aromas) and ageing/storage conditions (ageing aromas). 

There are clear sensory differences in wine aroma perception from different grape varieties. However, some of these differences are not really perceptible at pre-fermentative stages. Thus, most wine aroma compounds, including the varietal fraction that is generally conjugated in grapes, are produced or released during wine production and derived from microbial activity.

Nowadays, it is widely known that wine fermentation is not a single-species process, and the role of the different microbial wine-related species in wine production is in the spotlight of worldwide research [2]. Furthermore, following a holistic approach, wine grape microbiome could be related with the sensorial properties of wines [3]. This wine microbiome, which comes from vineyards, is therefore dependent on the geographical location, grape variety, climatic conditions and agronomical practices, establishing a new variable of the concept of *terroir* [4,5,6]. Recent results from Bokulich et al. [3] suggest that the microbial profile of grapes could predict the composition and abundance of certain wine impact metabolites. In some cases, these predictions, based in statistical models, could be confirmed with the metabolic characteristics of certain microorganisms previously studied as pure inoculum. However, there are many microbial species, both fermentative and dominant grape epiphytes, with potential incidence in wine flavour [7] but whose specific role in wine production is still poorly understood. 

In general, the fermentation-derived volatile compounds make up the largest percentage of the total aroma composition of wine. Alcoholic fermentation, mainly achieved by *Saccharomyces cerevisiae*, leads to formation of many alcohols and esters [8]. As a rule, most of the fermentative aroma compounds have high sensory thresholds and therefore do not individually contribute, in a significant way, to the distinctive aroma of wines. However, their combination establishes the basic matrix of wine aroma. On the contrary, most of the aroma impact compounds are present at low concentrations in grapes and wines, but because of their very low (ng·L^−1^) sensory thresholds they can have a major impact on the overall wine aroma. In many cases, the production of higher alcohols and esters are related with convoluted metabolic pathways of the central metabolism of yeasts, but, on the contrary, the release of some of the mentioned impact compounds (e.g., varietal terpenes or thiols) depends on the action of simple enzymatic steps. Due to the complexity of wine aroma, containing an enormous chemical diversity, the study of the entire volatile fraction of wine metabolome requires the use of multiple, complex and expensive chromatographic–spectrometric analyses. Thus, it is necessary to develop simpler and non-targeted methods in this field [9].

Lactic acid bacteria (LAB) are one of the most relevant groups of microorganisms in winemaking. They are responsible for the decarboxylation of malic acid to lactic acid in a process known as malolactic fermentation that usually takes place after the alcoholic fermentation. Malolactic fermentation is a process required for most red wines and some white wines; it makes wines more palatable by reducing the tart taste associated to malic acid, and provides additional advantages, like microbial stability and improved aroma complexity. *Oenococcus oeni* is a typical bacterial species isolated from spontaneous malolactic fermentation processes, although it is also used as a widespread starter culture for this purpose [10]. Some *Lactobacillus* strains and, in particular, the species *Lactobacillus plantarum*, have also been shown to be suitable to drive this process [11,12] and, in fact, there are some commercial malolactic starters of this species. Apart from its main sensory effect (depleting malic acid from wine) this secondary fermentation could modify the aromatic properties of wines by releasing notable concentrations of diacetyl (2,3-butanedione) and other carbonyl compounds obtained from citric acid, which contribute to the buttery aroma of wines. Many other biochemical reactions that occur at the same time also enhance wine aroma and quality. Esterase activity, methionine metabolism and some transformations involving glycosidases have also been demonstrated in LAB [13,14]. Nowadays, changes in wine flavour and aroma during malolactic fermentation that have been detected by sensory analyses, have now been identified at a molecular level [15]. However, more research should be made in this topic, considering also LAB species different to *O. oeni*.

Finally, ageing is a key step in the final flavour properties of some wines. It has been traditionally associated with red wines but, nowadays with more frequency, it is linked to white and rosé wines. During ageing process in oak barrels, woods transfer a series of wood-related aromatic substances to the wines. The volatile compounds extracted from wood are mainly furfural compounds, such as guaiacol, oak- or whisky-lactone, vanillin or syringaldehyde [16]. Apart from the role of wood during the ageing process, some microbial-derived compounds, such as polysaccharides, free amino acids, and peptides, could also contribute to the wine composition [17,18,19]. These compounds are derived from dead yeasts and bacteria cells (lees) that, during their lysis, could also liberate some active enzymes that continuously modify wine flavour during ageing. Thus, the intentional use of lees during wine ageing is a useful tool to modify wine composition [20,21,22]. However, long ageing times are not absent of spoilage risks, because of the action of ethanol-tolerant species such as *Brettanomyces bruxellensis* that can produce significant concentrations of ethylphenols, which are responsible for the unpleasant horsy and medicinal aromas [23].

In this context, this review aims to integrate the current knowledge about the role of microorganisms, mainly yeasts and LAB, in wine aroma and their potential use as biological tools to enhance wine quality. Furthermore, the conversion of aroma compounds by oral microbiota during wine consumption is considered. Finally, the advent of promising opportunities and challenges allowed by genomics on wine yeasts and bacteria research is also discussed. 

## 2. Fermentative Aroma Compounds

Wine volatiles are originated in part from grapes (varietal) and in part from fermentation processes. The major fermentation aroma constituents are ethanol, higher alcohols and esters. The pathways leading to the formation of flavour compounds that contribute to the overall taste of wine, such as the Ehrlich pathway for higher alcohols, or the enzymes responsible for the formation of esters, have been mainly studied in *Saccharomyces* species [24,25,26,27,28,29].

The yeast domain contains more than 2000 yeast species; several of them show potentially interesting traits for the food and beverage industries [30]. *Saccharomyces* yeast species are the most important yeasts involved in industrial-scale food fermentations. However, there are many other yeast species that are highly promising for flavour development and are still poorly studied. This work reviews natural yeast diversity in terms of aroma formation, with a particular focus on aromas relevant for wine quality. However, in both spontaneous and inoculated wine fermentations, the initial fermentation steps are carried out by a large number of non-*Saccharomyces* yeast genera (*Candida*, *Debaryomyces*, *Hanseniaspora*, *Hansenula*, *Kloeckera*, *Metschnikowia*, *Pichia*, *Lachancea*, *Brettanomyces*, *Kluyveromyces*, *Schizosaccharomyces*, *Torulaspora*, *Zygosaccharomyces* and *Saccharomycodes*) that contribute significantly to the overall aroma profile of the wine by producing flavour-active compounds [31,32,33,34]. During these initial steps, some of these species produce important aroma compounds, such as higher (fusel) alcohols derived from the Ehrlich pathway, ethyl esters and acetate esters in higher amounts than *Saccharomyces* species [35]. However, since general patterns of flavour contribution may be described at a species level, in most cases a great strain-dependence determines the amount of aroma compounds released, as occurs in *S. cerevisiae.* Additionally, results from Barbosa et al. [36] suggest that the use of mixed inoculation of non-*Saccharomyces* and *S. cerevisiae* cultures could modulate wine aroma not only by contributing individually, but also by changing the genomic expression patterns of *S. cerevisiae* due to their coexistence during wine fermentation. These changes affect, for example, the expression levels of different amino acid and ammonium transporters (*GAP1* and *AGP1* or *MEP1* and *MEP2* genes, respectively) involved in the production of important aromatic compounds, such as fermentative aromas (higher alcohols), and maybe they can also affect the production of other impact compounds, like varietal thiols.

### 2.1. Higher Alcohols and the Ehrlich Pathway Regulation

Wine fermentation is accompanied by the formation of the aliphatic and aromatic alcohols known as fusel or higher alcohols. Higher alcohol formation by yeasts is of great interest in the field of fermented beverages. Among them, medium-chain alcohols greatly impact the final flavour profile of alcoholic beverages, even at low concentrations. While fusel alcohols at high concentrations impart off-flavours, low concentrations of these compounds and their esters make an essential contribution to the basic matrix of flavours and aromas of wine. The aroma balance of these compounds in fermented foods and beverages is often used as an organoleptic fingerprint for the typicity of wines from different designation of origins, specific wines and brands. The main aliphatic alcohols include propanol, isoamyl alcohol, isobutanol and active amyl alcohol; and the main aromatic alcohols consist of 2-phenylethyl alcohol and tyrosol. Total higher alcohol concentrations range from 140 to 420 mg/L [37], where concentrations below 300 mg/L add a desirable level of complexity to wine, whereas concentrations that exceed this level can have a detrimental effect [38]. Quantitatively, the main higher alcohols in wine are isobutanol (9.2 mg/L), phenylethanol (6.1 mg/L) and isoamylalcohol (1.41 mg/L) [39], the other compounds being typically present at concentrations below their sensory thresholds.

The use of different yeast strains for wine fermentation contributes to significant variations in higher alcohol levels in wine [40,41]. The concentration of amino acids in the must, as the precursors for higher alcohols, also influences their production, where the total production of higher alcohols increases as concentrations of the corresponding amino acids increase [42]. As mentioned above, the use of mixed or sequential inoculations could change the higher alcohol profile of wines, depending on the strain used and the inoculation protocol implemented. *Lachancea thermotolerans, Hanseniaspora uvarum, Candida zemplinina*, *Saccharomycodes ludwigii* and *Pichia anomala* have been described as high fusel alcohols producers when used in single inoculations and in mixed fermentations with *S. cerevisiae*, generally with independency of the inocula ratio used. On the other hand, *Candida stellata* and *Zygosaccharomyces fermentati* species have been reported as low producers, when they were used as sole inocula and in co-inoculation with *S. cerevisiae* [33]. Regarding *Torulaspora delbrueckii*, contradictory results have been reported in this regard, since both increased and decreased levels of higher alcohols have been obtained depending on the strain and the inoculation protocol used [43,44,45]. Looking only into the strain variability, the production of higher alcohols in sequential inoculation of three industrial *T. delbrueckii* strains, Zymaflore^®^ Alpha (Laffort, Bordeaux, France), BIODIVA^®^ (Lallemand, Montreal, Canada) [44] and Viniferm NS-TD^®^ (Agrovin, Alcáazar de San Juan, Spain) [45], were evaluated, reporting that only the latter was able to reduce the sum of higher alcohols in sequential fermentations with *S. cerevisiae*. At this point, it should be studied in detail if these changes come from non-*Saccharomyces* metabolism, from modifications of the metabolic regulation of *S. cerevisiae* (because of their coexistence) or, presumably, as a sum of both factors.

**Figure 1:**
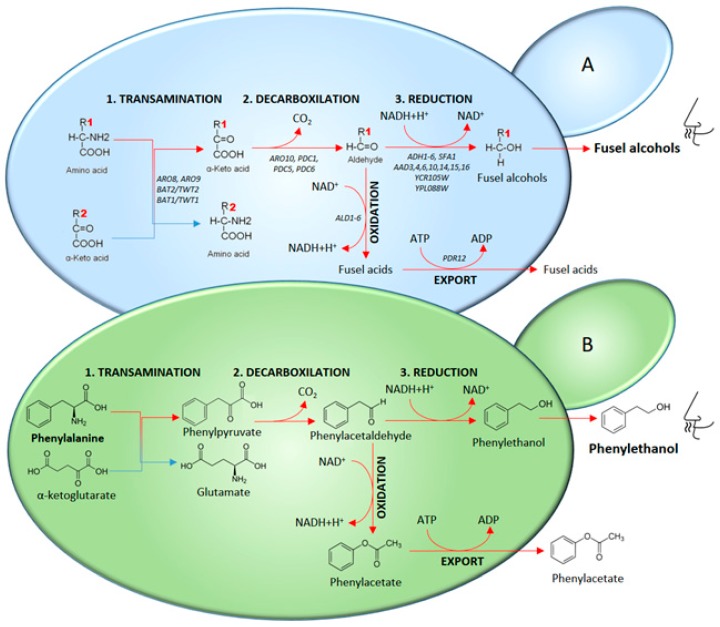
The Ehrlich pathway. Catabolism of aromatic amino acids (Phe, Tyr, and Trp), branched-chain amino acids (Leu, Val, and Ile) and sulphur-containing amino acids (Met) leads to the formation of fuse acids and fusel alcohols (**A**). Genes encoding enzymes involved in each step are shown. The catabolism of phenylalanine via the Ehrlich pathway leads to the formation of phenylethanol and phenylacetate (**B**).

Fusel alcohols are derived from amino acid catabolism via a pathway that was first described by Ehrlich [46]. Amino acids represent a major source of the assimilable nitrogen in grape must, and these amino acids are taken up by yeasts in a sequential manner. Amino acids that are assimilated by the Ehrlich pathway (valine, leucine, isoleucine, methionine and phenylalanine) are taken up slowly throughout the fermentation time. After the initial transamination reaction (Figure 1), the resulting α-keto acids are converted by yeast cells into fusel alcohols or acids via the Ehrlich pathway [47].

The transcriptional network regulation of the Ehrlich pathway remains to be fully explained. It is widely accepted that the amino acid metabolism in yeasts directly influences higher alcohol formation, especially the catabolism of aromatic and branched-chain amino acids. Amino acid catabolism is controlled by NCR (nitrogen catabolite repression), which is a complex regulation system that enables yeasts to select nitrogen sources that provide the best growth. NCR is mainly mediated by four transcription factors (GATA factors), as well as the regulatory protein Ure2p [48]. Once preferred nitrogen sources become limited in the medium, genes responsible for the utilisation of non-preferred nitrogen sources are gradually derepressed and NCR is lifted. 

During wine fermentation, some transcriptional studies showed that the enzymes involved in the main three steps (transamination, decarboxylation and reduction) of the Ehrlich pathway, which are codified by the genes *BAT2*, *PDC1* and *ADH1*, share similar expression profiles during alcoholic fermentation, while *BAT1*, which encodes a mitochondrial aminotransferase, is differently regulated [49]. Ehrlich pathway regulation seems to be very different depending on the growth phase, temperature and amino acid content, among others. For example, *BAT1* is preferentially expressed during the exponential growth phase, whereas *BAT2* is overexpressed during the stationary growth phase [50]. The overexpression of these two genes generates important increments of isobutanol and isoamyl alcohol but not of 2-phenyl ethanol.

**Figure 2:**
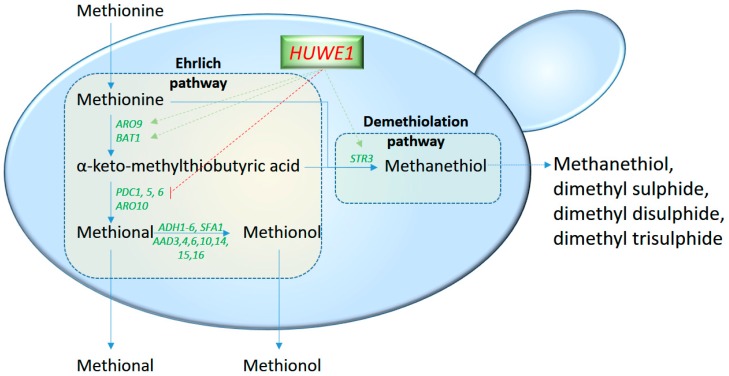
Overview of Huwe1-mediated regulation of the Ehrlich and demethiolation pathways. E3 ubiquitin-protein ligase Huwe1, a key constituent of the ubiquitin-proteasome system, increases the expression of the genes *ARO9* and *BAT1* of the Ehrlich pathway and of the demethiolation pathway gene *STR3* (green arrows), but suppresses (red arrow) the expression of Ehrlich pathway genes *PDC1*, *PDC5*, *PDC6*, *ARO10* in *S. cerevisiae* [51].

In conclusion, further studies should be conducted to better understand the relation between expression profiles and higher alcohol production. In this way, the E3 ubiquitin-protein ligase Huwe1 has been shown to be involved in Ehrlich pathway regulation [51]. The Ehrlich pathway works simultaneously with the demethiolation pathway as two competing branches that convert amino acid into alcohols (Figure 2). Overall, Huwe1, a key constituent of the ubiquitin-proteasome system, increased the expression genes *ARO9* and *BAT1* of the Ehrlich pathway and the demethiolation pathway gene *STR3*, but suppressed the expression of the Ehrlich pathway genes *ARO10*, *PDC1*, *PDC5* and *PDC6* in *S. cerevisiae.* Controlling both pathways offers considerable potential for industrial applications, including alcohols overproduction and flavour-quality control. However, how to regulate the Ehrlich and demethiolation pathways in industrial processes is still not applicable [51].

In this context, it should be taken into consideration that the Ehrlich pathway and the specific enzymes responsible for later ester synthesis, are also present in non-*Saccharomyces* yeasts. Furthermore, as occurs in *S. cerevisiae*, the enzymes of the Ehrlich pathway (such as Aro10p) are also broad-substrate specific, resulting in the formation of a broad range of fusel alcohols even if only one amino acid is provided as sole nitrogen source [47]. However, different profiles and amounts of higher alcohols are produced by different yeast species in similar fermentative conditions, indicating that the mechanisms involved in the regulation of the Ehrlich pathway are also diverse in non-conventional yeasts compared to *Saccharomyces* species [35]. Therefore, these mechanisms should be studied in detail to better understand the contribution of non-*Saccharomyces* yeasts to wine flavour and to improve the exploitation of their metabolism in wine fermentations.

In summary, the higher alcohols derived from the degradation of amino acids via the Ehrlich pathway have direct impacts on the aroma of wine, but they are often precursors to the formation of an important family of wine aromas: the esters. 

### 2.2. Esters

Wine contains several groups of compounds that are formed through the same biosynthetic route, and that in addition can share the same aromatic and chemical properties. These groups of compounds tend to act collectively exerting a concerted (additive) effect on wine aroma [52]. Esters, such as ethyl esters of branched fatty acids (ethyl 2-methylbutyrate, ethyl isobutyrate, ethyl isovalerate, ethyl 2,3 and 4-methylpentanoates) are among these compounds. 

Esters are formed during alcoholic fermentation, malolactic fermentation and ageing. There are many factors that influence the types and quantities of esters present, and, although not all of them are positive contributors, they constitute a major group in wine. Esters present in wines can have different origins: grapes, yeasts and bacteria. The esters from grapes are relevant in wines only in particular cases [53], the most important esters for wine aroma being synthetised by yeasts.

Esters are metabolic by-products of yeasts for three main reasons. First, they are usually less toxic than their correspondent alcohol or acidic precursors (a detoxification mechanism); second, they are insect attractants, serving as a mechanism for yeast spread; finally, their synthesis serves as a mechanism for the regeneration of free Coenzyme A from its conjugates. During wine fermentation, maximum concentration of esters is obtained when yeasts reach the stationary growth phase [54].

**Figure 3:**
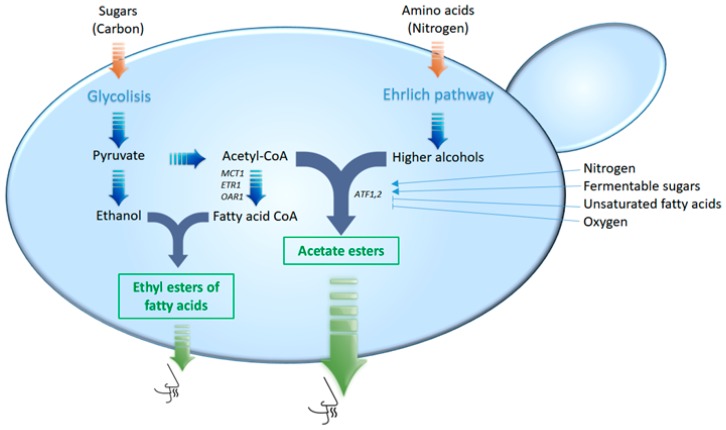
Simplified metabolic map of ester formation. Genes that codify enzymes involved in ester formation are indicated. The substrate availability depends mainly on carbon, nitrogen, and fatty acid metabolism, while the ester synthase activity is mainly determined by the activity of the corresponding genes. Thus, factors that affect yeast metabolism or ester synthase gene regulation will influence ester synthesis.

Volatile esters have a higher impact in wine aroma compared to higher alcohols, although esters are present in small quantities, in orders of few mg/L, and their concentration diminishes by chemical hydrolysis during ageing [55]. These compounds are perceived as generically fruity or floral. However, if they are present in too high amounts, they can mask varietal aromas, decreasing wine complexity (wines containing more than 90 mg/L of ethyl acetate or 200 mg/L of total esters are considered defective). There are two main classes of esters formed in wine: the ethyl esters of fatty acids and the acetate esters of higher alcohols. Ethyl esters are formed between ethanol and fatty acids or non-volatile organic acids (Figure 3) and contribute to the wine aroma with low prominent odours reminiscent to candle wax or soap.

Acetate esters, more important for wine aroma than ethyl esters of fatty acids, derive from the conjugation of an alcohol with an acid. Apart from ethanol, common alcohols found in esters derive from the degradation of amino acids via the Ehrlich pathway, as stated before. The most significant are isobutyl acetate (fruity aroma), isoamyl acetate (banana), and 2-phenylethyl acetate (flowery aroma), which result from the esterification of their corresponding higher alcohols: isobutanol (derived from the amino acid valine), isoamyl alcohol (derived from leucine), and 2-phenylethanol (derived from phenylalanine), respectively [29,56]. 

Several different enzymes are involved in the formation of esters [57], the best characterised ones being the alcohol acetyl transferases I and II, which are encoded by the genes *ATF1* and *ATF2*, respectively [58,59]. These enzymes transfer the acyl group (acid group) of an acyl-CoA conjugate to a receptor higher alcohol (Figure 3). The most common acyl-CoA molecule found in yeast is acetyl-CoA and, thus, the most common esters are acetate esters. Atflp and Atf2p are partially responsible of isoamyl acetate and ethyl acetate production [59]. Differences found among three *Saccharomyces* species (*S. cerevisiae*, *S. kudriavzevii* and *S. uvarum*) during the aroma-active acetate ester formation have been indicated to be due, to some extent, to the distinct properties of Atf enzymes [56].

Since differences in ester-related enzymes have been described among *Saccharomyces* species, a considerable diversity is expectable in other non-*Saccharomyces* yeasts that, in some cases, have been described as useful tools to increase ester concentration in wines [60]. As an example of this contribution, it should be highlighted the great ability of *M. pulcherrima* to produce high concentrations of pear-associated esters, such as ethyl octanoate, in co-inoculation with *S. cerevisiae* [61]. Another example is the capability of *Hanseniaspora guilliermondii* and *H. vineae* to produce 2-phenylethanol and 2-phenylethyl acetate, in both single inoculation and co-inoculation with *S. cerevisiae,* which are aromatic compounds linked with rose, flowery and honey descriptors [62,63,64,65]. In the case of *Saccharomyces* species, considerable differences have been reported in ester production profiles between *S. cerevisiae*, *S. bayanus* and *S. japonicus* strains [60]. It is of interest to study the reasons of the enhanced/reduced ester release, focusing on gene structure, transcriptional regulation and enzyme kinetics in wine fermentation conditions. For that purpose, aromatic differences in closely-related yeast species and strains, such as *Saccharomyces* spp., bring to us the opportunity to finely study the genomic and transcriptomic differences in similar orthologous genes that explain this variability.

The net accumulation of esters in wine is determined by the balance between the ester-synthesizing enzymes and esterases from yeasts (responsible for cleavage and in some cases, formation of ester bonds) [66]. Although extracellular esterases are known to be present in *S. cerevisiae* [67], the situation for non-*Saccharomyces* needs further investigation.

Different studies demonstrated that wine LAB exhibit enzyme activities that can augment the ethyl ester content of wine [68,69]. However, while the esterases from yeasts have been widely studied, esterase activity for wine-related LAB is not well documented [70,71]. For example, *O. oeni* produced significant levels of ethyl hexanoate and ethyl octanoate following growth in an ethanolic test medium, and also esterified 1-propanol to produce propyl octanoate [72]. However, the concentrations of some of these compounds appear to be influenced by the LAB species and the strain used, reflecting a degree of diversity among strains of the same species [68]. Intracellular esterases from *O. oeni* and *Lactobacillus hilgardii* were characterised under wine-like conditions. Both esterases were stable and retained activity under conditions that would be encountered in wine. They have the potential to reduce short-chain ethyl esters such as ethyl acetate [70]. Among wine LAB, besides *O. oeni*, *L. plantarum* strains are also used as malolactic starters. *L. plantarum* is a good source of esterase enzymes; in fact, some esterase proteins have been purified and characterised in this species [73]. However, there is still limited information on the function of genes coding for esterases and their potential contribution to wine aroma. Lp_1002 was the first arylesterase described in a wine LAB. This *L. plantarum* esterase possessed suitable biochemical properties to be used during winemaking (resistance to ethanol, sodium metabisulphite, and tartaric, lactic and citric acids, with only malic acid slightly inhibiting Lp_1002 activity) [74]. 

## 3. Wine Microorganisms and Varietal Aroma Compounds

### 3.1. Production of Volatile Sulphur Compounds

Among the volatile metabolites released by yeasts involved in the aroma of wine, the sulphur-containing compounds are of great importance, causing a strong influence in wine organoleptic properties because of their very low detection thresholds [75]. Sulphur compounds have a considerable diversity, showing different sensory properties depending on the position of the sulphur atom in the molecule and their concentration [66]. Although most of these sulphur compounds contribute negatively to wine quality, some of them have a positive effect on the aromatic properties of wines [76].

The presence of sulphur compounds in wines has two main origins: (1) the non-enzymatic process that includes chemical reactions (such as photochemical) of sulphur compounds during winemaking and storage; and (2) the enzymatic process that includes the degradations of sulphur-containing amino acids during fermentation by yeasts [75] and by lactic acid bacteria [77]. 

#### 3.1.1. Undesirable Sulphur Compounds

The highly volatile sulphur compounds are present in wine in concentrations above their perception threshold and they possess strong off-flavours, often referred to as reductive aromas, identified as rotten eggs, garlic, onion and cabbage odours [78]. However, small amounts of these negatives aromas can be perceived as beneficial to aromatic complexity [79]. The most important sulphur compounds with a negative impact in the sensory quality of the wine are: hydrogen sulphide, methanethiol and dimethyl sulphide [79].

##### Hydrogen Sulphide

The presence of H_2_S in wine is an important problem for the wine industry because it imparts undesirable off-flavours like sulphurous or rotten egg aromas as well as due to its low perception threshold (10 to 80 μg/L) [80]. As a result of its high reactivity, H_2_S can take part in many reactions which generate compounds that can also affect the flavour of wine. For example, ethanethiol (a strongly unpleasant odour) can be formed by the reaction of H_2_S and the ethanol produced during alcoholic fermentation [81].

There are several factors that are implicated in the appearance of H_2_S: high residual levels of elemental sulphur, presence of sulphur dioxide, presence of sulphur organic compounds, high concentration of amino acids like threonine, methionine or cysteine, and nitrogen limitation [82]. Another source of sulphur is its supply against microbial growth, such as potassium metabisulphite [79]. 

In addition of these sources, the major H_2_S production occurs during the biosynthesis of the sulphur-containing amino acids methionine and cysteine [83] from inorganic or organic sulphur sources [84]. Thus, wine yeasts strongly affect H_2_S production during must fermentation, along with nitrogen limitation and sulphur composition of grape must [85]. The reasonable concentration range of H_2_S reported in wines is from trace to 80 μg/L.

**Figure 4:**
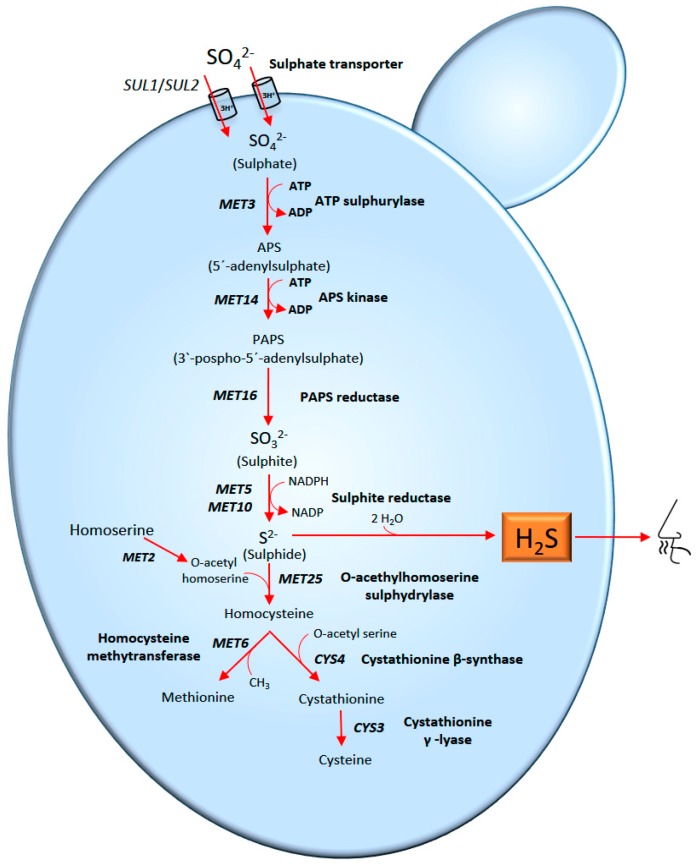
The first step of the pathway consists of the sulphate uptake through two specific permeases in co-transport with 3H^+^. Then, sulphate is reduced to sulphide in several steps using the enzymes ATP sulphurylase and sulphite reductase. The sulphide afterwards combines with *O*-acetylserine to form cysteine or *O*-acetylhomoserine to form homocysteine, which then can be transformed to methionine. H_2_S derives from the S^-2^ ion, an intermediate in the reduction of sulphate or sulphite for the synthesis of these amino acids.

In *S. cerevisiae*, H_2_S is produced by the sulphate reduction sequence (SRS) pathway (Figure 4). The SRS pathway is induced when there is a metabolic demand for cysteine and methionine, which are usually limited in must [66,84,86,87,88]. When there are insufficient nitrogen sources, the precursors for these amino acids (*O*-acetylserine and *O*-acetylhomoserine) will also be limited. The lack of precursors supposes that the pathway stops at sulphide. The sulphide, which cannot be sequestered by *O*-acetyl serine and *O*-acetyl homoserine to form methionine or cysteine, produces H_2_S. This volatile gas can accumulate and diffuse into the wine [66].

The entire sulphur metabolism pathways are also regulated through several mechanisms controlled by the intracellular concentration of cysteine. These mechanisms involve the transcription of genes of sulphate assimilation and of sulphur-containing amino acid synthesis, which are mainly regulated by *MET4* [89] and *GCN4* genes [90].

Several genetic approaches have been developed in order to decrease H_2_S production in wine. Spiropoulus and Bisson [82] showed that overexpressing the *MET17* gene (encoding *O*-acetylserine/*O*-acetylhomoserine sulphhydrylase) in *S. cerevisiae* significantly reduced H_2_S production in wine. In addition, overexpression of the *CYS4* gene, which encodes cystathionine β-synthetase, showed a reduction of H_2_S production [91]. However, these approaches involve the use of genetically modified yeast strains, which is rejected in wine industry.

Another strategy to reduce H_2_S production is the application of non-conventional yeasts in winemaking. Sulphite reductase activity, one of the main enzymatic activities responsible for H_2_S production, is a rare feature among the majority of non-*Saccharomyces* species [7], since only species from *Hanseniaspora* genus (mainly *H. osmophila* and *H. opuntiae*) had quite high sulphite reductase activity among the 15 species tested. In addition, some *T. delbrueckii* strains, apart from *S. cerevisiae*, had certain H_2_S production ability. However, as occurres in *S. cerevisiae*, a great strain-dependent behaviour exists in other wine related yeast species, such as *Dekkera, Lachancea, Hanseniaspora,* and *Metschnikowia* [7,85]. 

##### Dimethyl Sulphide and Methanethiol

Dimethyl sulphide is a reductive aroma, although at low concentrations it is described as having blackcurrant, red fruit and truffle aromas and it is considered to enhance the *bouquet* in some wine styles [79]. At high concentration, dimethyl sulphide can impart canned corn, asparagus or vegetal aromas [75]. With a perception threshold of 25 μg/L, this volatile compound can be found in most wine varieties, in concentrations ranging from 1.4 to 61.9 μg/L, obtaining the highest values during ageing [66,92]. 

Methanethiol (or methyl mercaptan) is characterised by odours of rotten egg, sewage and rubber. Its perception threshold is 0.3 μg/L and it can be found in concentrations from 0.7 μg/L in normal wines to 2.1 in reduced wines [66]. It is produced during the first stages of fermentation, when yeasts assimilate sulphur-containing nutrients [93]. Methionine can produce methanethiol through transamination to form the α-keto-γ-(methylthio)-butyrate, and through demethiolase activity [94], and it seems to be formed by a methionine γ-lyase in yeast [93].

As H_2_S, wine redox potential (E_H_) during bottle storage is determining for dimethyl sulphide and methanethiol concentrations. In the presence of oxygen, for example during racking and at a high E_H_, methanethiol is oxidised to dimethyl sulphide. Since the perception threshold of dimethyl sulphide is 60 times higher than that of methanethiol, the off-flavour disappears. However, if the oxygen is consumed, the E_H_ diminishes and the reaction can be reversed [95].

Wine LAB may also transform methionine into sulphur compounds that generally impact sensory quality. In laboratory cultures, LAB have been found to metabolise this amino acid, resulting in the formation of characteristic aromas that contribute to the aromatic complexity of wine; some examples include methanethiol, dimethyl disulphide, 3-(methylsulphanyl)propan-1-ol, and 3-(methylsulphanyl) propionic acid [77]. The reduction of methanethiol is the last stage in the enzymatic synthesis of methanethiol from methionine. Vallet et al. [96] purified the alcohol dehydrogenase enzyme involved in this conversion. All strains of lactobacilli and, specially, *O. oeni* form significant quantities of methanethiol and dimethyl disulphide. Only 3-(methylsulphanyl)-propionic acid is systematically formed in significant amounts during malolactic fermentation in red wine, with a potential organoleptic impact when final concentrations exceed the perception threshold. In wine, the relevant aroma descriptor is red-berry fruit [77]. The first reaction of this metabolic pathway in *O. oeni* is catalysed by an aminotransferase similar to the aromatic amino acids and aspartate aminotransferase described in other LAB [15]. This is the key reaction in *O. oeni* methionine metabolism, producing an intermediate compound, 2-oxo-4-(methylthio)butyric acid, which is reduced to methional, another important intermediate. 

#### 3.1.2. Desirable Sulphur Compounds

On the other hand, less volatile compounds with a higher molecular weight are usually found at low concentrations (below their threshold value). They generally impart box tree, passion fruit and grapefruit aromas [84]. Among them, there are compounds called “tropical volatile thiols”, 4-mercapto-4-methylpentane-2-one (4-MMP), 3-mercapto-hexanol (3-MH) and 3-mercaptohexylacetate (3-MHA) being the most important sulphur compounds in the varietal aroma of white wines [97], with detection thresholds of 3 ng/L, 60 ng/L and 4 ng/L, respectively. Other related thiols, which are contributors to the characteristic flavour of this kind of wines are 4-mercapto-4-methyl-pentan-2-ol (4-MMPOH) and 3-mercapto-3-methylbutan-1-ol (3-MMB) [76]. Below, we will focus on 4-MMP, 3-MH and its acetate ester 3-MHA, as the main contributors to the varietal thiol profile of some white wines [66].

As varietal odours, thiol aromas are relatively unexpressed in grapes but developed during alcohol fermentation [98]. It has been shown that 4-MMP and 3-MH exist in grapes in their non-volatile precursor forms, conjugated with cysteine [99,100] or glutathione [101]. Carbon-sulphur lyase enzymes of yeasts are necessary to cleave the cysteine-glutathione conjugated precursor, releasing the correspondent volatile thiols [102]. The cystenilated forms are generally more abundant than the gluathionylated forms in must [103]. 

Although thiol production depends on different factors (temperature during fermentation, additions of nutrients, prefermentative operations, thiol precursors concentrations in grapes, and oxygen, phenol or sulphur dioxide content), the yeast strain used to conduct the fermentation is one of the most important factors that affect thiol releasing [83,104]. 

After alcoholic fermentation, thiols are chemically unstable in the presence of oxygen, so the storage and ageing conditions are important in the final thiol aroma of wines. However, the absence of oxygen can produce reduced dominant odours [103]. As a result, it is necessary to achieve suitable methods of storage and ageing to preserve the aroma and control the oxidation reactions.

#### 3.1.3. Metabolic and Gene Regulation

The *S. cerevisiae* genes required for the conversion of conjugated precursors into varietal thiols in wine have been identified [105]. The uptake of the precursors is mediated by general amino acid transporters, being the *GAP1* and *OPT1* transporters responsible for the uptake of the major part of the cysteinylated and glutathionylated precursors, respectively [106,107].

Once inside the cell, the cysteinylated precursors are cleaved by a carbon-sulphur β-lyase enzyme. Four genes (*BNA3*, *CYS3*, *GLO1* and *IRC7*) identified as β-lyase enzymes influence the release of volatile thiol 4MMP [108] being Irc7-p the main enzyme responsible for its production [109]. However, most strains of *S. cerevisiae* present a 38-bp (base pair) deletion in the *IRC7* sequence that encodes for a less functional enzyme [109,110]. *STR3* β-lyase is also responsible for thiol release, with a remarkable incidence in 3MH but with a low specific activity [111]. 

**Figure 5:**
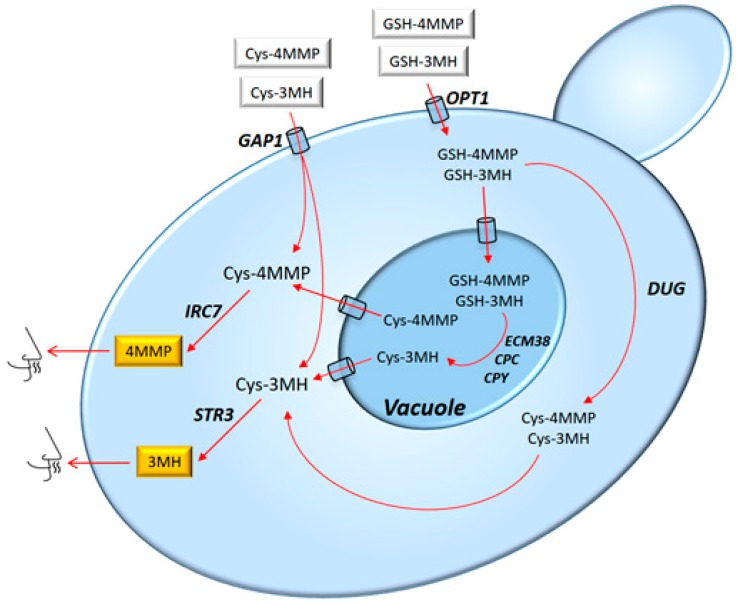
Metabolic and gene regulation of the production of varietal thiols. The uptake of the precursors is mediated by general amino acid transporters. Gap1p and Opt1p transporters are responsible for the uptake of the major part of the cysteinylated and glutathionylated precursors, respectively. Once inside the cell, the cysteinylated precursors are cleaved by a carbon-sulphur β-lyase enzyme. Glutathionylated precursors, which enter the cell through Opt1p, are not cleaved directly, but they are degraded to the cysteinylated form as an intermediate in a multi-step pathway.

Regarding the glutathionylated precursors, which enter the cell through Opt1p, they are not cleaved directly, but they are degraded to the cysteinylated form as an intermediate in a multi-step pathway. The genes *DUG1, DUG2, DUG3, CPC, CPY* and *ECM38* seem to be implicated in this process [83,107]. The *CIS2* gene, encoding γ-glutamyl transpeptidase, is also required for the conversion of glutathione precursors to volatile thiols [105] (Figure 5).

One of the most important regulation controls of thiol releasing pathways is NCR. Through NCR, the preferred nitrogen source exerts a negative effect on the expression of many genes involved in the use of non-preferred nitrogen sources. Regarding the thiol release process, NCR has incidence in both conjugated precursors transport [106] and in the later precursor cleavage [112], but with notable differences among strains. Thus, the nitrogen metabolism and volatile thiols release are tightly related. Therefore, it is important to carry out suitable nitrogen nutrition during alcoholic fermentation to achieve the maximum aromatic potential of grape varieties.

Although *S. cerevisiae* is the main yeast in the fermentative process, it is only able to release as volatile thiols around 10% of the precursor originally present in the must [113,114]. This low efficiency is attributed to the NCR, which affects transport genes (such as *GAP1*) [106] and genes involved in precursors cleavage (such as *IRC7*) [112]. Thus, the application of non-conventional yeasts in winemaking is an alternative to improve the volatile thiol production in wine.

Results from Zott et al. [115] evaluating the thiol release ability of 15 non-*Saccharomyces* yeast strains confirm that non-*Saccharomyces* yeasts can contribute positively to volatile thiol release from their cysteinylated precursors, but generally with a higher incidence in 3-MH. Although volatile thiol production appeared to be low for most of the non-*Saccharomyces* species tested, some *M. pulcherrima*, *T. delbrueckii* and *L. thermotolerans* strains showed a high capacity to release volatile thiols, mainly 3-MH, in single inoculations. At this point, Anfang et al. [116] also reported a significant enhancement of 3-MHA production by *Pichia kluyveri* in co-fermentation with *S. cerevisiae.* In addition to these species, Belda et al. [7,110] evidenced a remarkable β-lyase activity from cysteinylated precursors in *T. delbrueckii, Kluyveromyces marxianus* and *Meyerozyma guilliermondii*. 

Renault et al. [117] reported the ability of an industrial *T. delbrueckii* (Zymaflore^®^ Alpha^TDn.sacch^), strain to release 3-MH in co-culture fermentation with *S. cerevisiae*, but not 4-MMP. In addition, their results suggest that *T. delbrueckii* is not able to assimilate the cysteinylated precursors of thiols, which are the most abundant forms for 4-MMP in grape musts. Recent results from our group partially contradict these conclusions, since a great 4-MMP production has been observed using another industrial *T. delbrueckii* strain (Viniferm NS-TD) in sequential inoculation with two different *S. cerevisiae* strains (both with full-length and short alleles of the *IRC7* gene), reaching values up to 70 ng/L of 4-MMP (ten times higher than its threshold). These results suggest that the intraspecific diversity concerns not only the precursor-cleavage β-lyase enzymes, but also other relevant genes, such as those encoding oligopeptide and amino acid transporters.

### 3.2. Wine Yeast and Bacteria Production of Monoterpenes

The aromatic profile of many wines depends on the varietal compounds of the grapes that have been employed in their production. These varietal compounds can be present in grapes as free volatile compounds and, in much higher concentrations, as aroma precursors [118]. Among them, non-volatile sugar-bound conjugates are odourless molecules which represent a natural reservoir of odorant compounds in wines, which can be naturally and slowly released during wine ageing or intentionally released by using oenological enzymes during winemaking. The volatile compounds that can be released from glycosidic aroma precursors are mainly terpenes, C13 norisoprenoids, benzenic derivatives, volatile phenols and C6 compounds [118]. These compounds are generally potent flavour compounds characterised by low odour thresholds and interesting sensory properties [119]. 

For example, terpenoids include a diverse family of varietal compounds derived from isoprene (2-methyl-1,3-butadiene), involved in the flower and fruit aroma of wines [83], being the main descriptors of white wine varieties like Muscat, Riesling or Albariño [120]. Terpene compounds belong to the secondary plant constituents and are biosynthesised from acetyl-CoA, taking part as intermediates the isopentenyl diphosphate and dimethyl allyl diphosphate [119]. *S. cerevisiae* lacks enzymes with monoterpene synthase activity, so they cannot produce monoterpenes efficiently; only a few natural strains are able to produce small amounts of monoterpenes [24].

Monoterpenoids consist of two isoprene units (10-carbon compounds) and have a strong sensory potential. They are produced from geranyl pyrophosphate by plants, algae, filamentous fungi and yeasts [121]. Three types of monoterpenes exist in plant tissues: first, free aroma compounds like linalool, geraniol, nerol, citronellol or myrcenol, and also several monoterpene ethyl ethers and acetate esters are present in wine; second, the polyhydroxylated and odourless forms of the monoterpenes, called polyols, which can be hydrolysed releasing pleasant flavours; and third, the glycosidically conjugated forms of the monoterpenes, which do not contribute to the grape juice aroma [122]. In general, most monoterpenes are in a glycosidically form, but only a small part can be found as free forms [123]; however, this distribution can vary amongst different grape varieties. 

The glycoside moiety of terpene precursors can be linked to different sugars: the monosaccharide β-d-glucose and the disaccharides 6-*O*-α-l-rhamnopyranosyl-β-d-glucopyranose, 6-*O*-α-l-arabinofuranosyl-β-d-glucopyranose, 6-*O*-β-d-xylopyranosyl-β-d-glucopyranose and 6-*O*-β-d-apiofuranosyl-β-d-glucopyranose [124,125]. Free terpenes can be released from terpene glycosides by acidic hydrolysis. Different monoterpenes, such as linalool, nerol or geraniol, are produced at different pH values in grape must. This spontaneous process can entail molecular rearrangement, transforming monoterpenes in other compounds [122]. Besides pH-dependent formation of terpenes (a relatively slow process), enzymatic hydrolysis of the terpene glycosides is a biological process of great importance in the production of volatile terpenes [126]. These terpenoids can be released by glycosidase enzymes produced by grapes, yeasts and bacteria, contributing to wine aroma and flavour [127].

**Figure 6:**
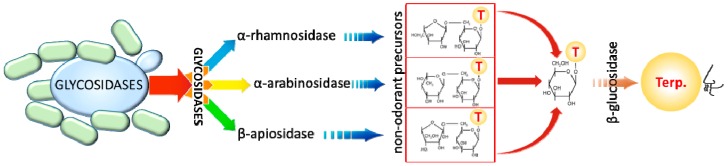
Release of terpenes from their correspondent non-odorant precursors by glycosidases of microbial origin.

The mechanism for the liberation of sugar-bound monoterpenes occurs in two sequentially steps: first, the enzymes α-l-rhamnosidase, α-l-arabinofuranosidase, β-d-xylosidase or β-d-apiosidase cleavage the bound between the terminal glucose and rhamnose, arabinose, xylose or apiose, respectively; then, a β-d-glucosidase hydrolytic enzyme releases the correspondent monoterpenol [128] (Figure 6). Thus, yeast glycosidase enzymes have a great relevance in winemaking because they are responsible for revealing the aromatic terpene compounds from their glycosilated precursors during alcoholic fermentation.

β-Glucosidase is a widespread activity among wine yeast species, the other glycosidase activities being restricted to just a few of them [7]. In the case of *S. cerevisiae,* a poor production of α-l-rhamnosidase, α-l-arabinofuranosidase, β-d-xylosidase and β-d-apiosidase activities has been reported [129]. Regarding its β-glucosidase activity, contradictory results have been reported, suggesting a great strain-dependent behaviour. In this sense, the existence of a gene in *S. cerevisiae* genome coding for an authentic functional β-glucosidase is not clear, although it seems that a few strains have one pertaining to the *BGL* genes family [126,130]. Nevertheless, winemaking conditions with low pH values, an increasing ethanol level and, specially, high glucose concentrations that exert catabolic repression on these enzymes, suppose a loss of stability of glycosidase enzymes in *S. cerevisiae* strains, causing a very limited activity in wine fermentation conditions [131,132,133].

On the other hand, it has been described the ability of *S. cerevisiae* to transform terpenol compounds produced by their glycosidase enzymes in other volatile compounds in a strain-dependent process, modifying the terpenic profile of the wine (e.g., geraniol was transformed into geranyl acetate, citronellyl acetate and citronellol) [119].

In this context, due to the low monoterpenyl glycosidase activity of most *S. cerevisiae* strains, a common strategy to improve the glycosylated terpenes hydrolysis is the addition of enzyme preparations, mainly obtained from *Aspergillus* spp. [83,127]. However, this process can increase industrial costs and these enzyme preparations tend to have low specificity, so it might induce secondary reactions which might negatively affect wine flavour [134].

Engineering *S. cerevisiae* wine strains by expressing enzymes able to hydrolyse glycosylated terpenes is another strategy to improve the terpene aroma profile in wines. Margolles-Clark et al. [135] addressed two decades ago the development of a recombinant *S. cerevisiae* strain encoding *ABFI* (α-l-arabinofuranosidase) and *BXLI* (β-d-xylosidase) genes from *Trichoderma reesei*, since the existence of an encoding gene for these glycosidase activities in *S. cerevisiae* genomes is still not clear. Zietsman et al. [136] also developed a recombinant *S. cerevisiae* strain co-expressing an α-l-arabinofuranosidase from *Aspergillus awamori* and a β-glucosidase from *Saccharomycopsis fibuligera,* which produced higher concentrations of volatile terpenes, supposing higher floral and fruity aromas in wine fermented with this yeast strain. Another strategy is the development of recombinant *S. cerevisiae* strains expressing enzymes from plants (such as *Vitis vinifera)* with monoterpene synthase activity, which catalyse the conversion of the universal precursor geranyl diphosphate to monoterpenes [83]. 

Regarding non-*Saccharomyces* species, they could have an important role in volatile free terpene production through their glycosidase activity. Results from Belda et al. [7] showed that many non-*Saccharomyces* species are able to produce not only β-d-glucosidase, but also high levels of β-d-xylosidase and α-l-arabinofuranosidase, the latter being the less distributed activity among yeasts and completely absent in fermentative yeast species such as *T. delbrueckii* and *Z. bailii*. Additionally, these two species are able to produce certain glycosidase activities, like β-d-glucosidase or β-d-xylosidase, but it has been observed a high glucose-dependent repression, as occurred in *S. cerevisiae* [126,137].

Yeasts of the *Hanseniaspora* genus isolated from grape must show a great β-d-glucosidase activity [138], and, in the case of *H. uvarum*, some strains are able to produce β-glucosidase enzymes with no repression by glucose and low pH values [131]. Similarly, some *Wickerhamomyces anomalus* strains, which show remarkable production levels of β-d-glucosidase, β-d-xylosidase and α-l-arabinofuranosidase enzymes [7], have also lower repression levels by pH, glucose and ethanol for its glycosidase activities [137,139]. 

Other studies also reported a high β-d-glucosidase activity from *M. pulcherrima*, *M. guillermondii*, *Issatchenkia terricola*, *Debaryomyces* spp. and different basidiomycetous species such as *Rhodosporidium toruloides* and *Cryptococcus amylolentus* [7,33,132,140], but their activity and applicability in wine conditions needs to be studied in depth. Since most glycosidase-encoding genes are repressed by glucose, some of the above-mentioned approaches were developed using enzymatic extracts or immobilised enzymes. Thus, the use of yeasts to enhance varietal terpenes in real industrial conditions needs to be further investigated, since it could be of great interest to improve the quality of neutral-aromatic grape varieties.

In comparison with yeasts, the performance of LAB on the hydrolysis of glycosylated derivatives in grape must and wine is poorly understood. These diverse reactions have attracted considerable interest, as they may clarify the role of bacteria in the sensory changes observed during malolactic fermentation, which contribute to wine colour and aroma. Some transformations involving glycosidases have been demonstrated in *O. oeni* [141]. The release of variety-specific volatile compounds has been observed for Tannat, Chardonnay and Muscat wines comparing the impact of several malolactic cultures [142,143,144]. The grape variety aromas released by bacteria vary widely, depending on the strains and terpene substrates involved. Boido et al. [142] suggested that the aromatic aglycones released are further metabolised, unless they become trapped by macromolecules, such as bacterial exopolysaccharides. Recently, some authors have evidenced that *L. plantarum* shows a different enzymatic profile compared to other LAB species, which suggests that this species could play an important role in the wine aroma profile [121,145]. Recently, Iorizzo et al. [146] identified different *L. plantarum* strains which exhibited a strong β-glucosidase and α-glucosidase activities in winemaking conditions. Furthermore, the ability of *L. plantarum* strains to release odorant aglycones was first demonstrated with a commercial octyl-β-d-glucopyranoside. Finally, in order to take a step forward in the ability of these bacteria to release aroma compounds in wines, a natural precursor extract obtained from white grapes was incubated in the presence of each strain. Interestingly, *L. plantarum* M10 strain released considerable amounts of important odorant compounds with low odour thresholds and flowery-citric aroma nuances in wines, such as the terpenes limonene and linalool, among others [146], which again suggested a relevant impact of *L. plantarum* on the production of important odorant molecules in wine.

## 4. Citric Acid Degradation by Lactic Acid Bacteria and Its Impact on the Aromatic Quality of Wine 

Citric acid, one of the acids present in both grapes and must, is generally found at lower concentrations (0.1–1 g/L) than major organic acids such as tartaric (2–8 g/L) and malic acids (1–7 g/L). Wine LAB are able to metabolise citrate, obtaining acetic acid and diacetyl as end products, and therefore impacting the aromatic quality of wine [147]. 

**Figure 7:**
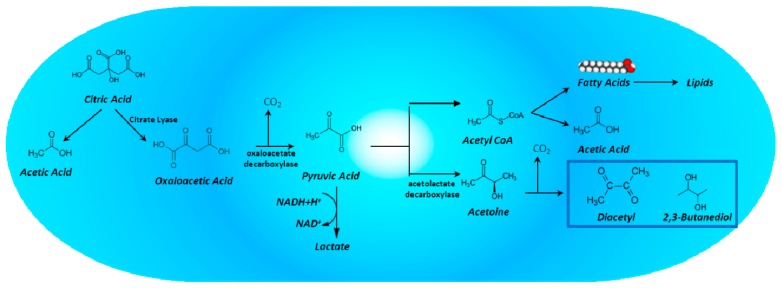
Schematic representation of citric acid degradation pathway by lactic acid bacteria.

Like other LAB, *O. oeni* does not use citrate as a sole carbon source but metabolises it together with glucose. The resulting biomass is greater than the one produced when this bacterium is grown in the presence of glucose alone. After being transported to the intracellular environment, citrate is converted into a mixture of lactate, acetate, diacetyl, acetoin and 2,3-butanediol (Figure 7). The bacterium breaks down the citrate into oxaloacetic acid in a reaction catalysed by citrate lyase. This acid is converted by oxaloacetate decarboxylase into pyruvate, which is mostly reduced to lactate in the presence of NADH. Some pyruvate, however, is converted by acetolactate decarboxylase to acetolactic acid, giving rise to acetoin and 2,3-butanediol following decarboxylation. The chemical oxidation of acetoin, in turn, yields diacetyl. The precursor of diacetyl (and acetoin), α-acetolactate, is also an intermediate in the biosynthesis of the amino acids valine and leucine. The degradation of citric acid by LAB automatically leads to an increase in volatile acidity in wine (as an average, 1.2 molecules of acetic acid are produced from each molecule of citric acid). However, due to the small quantities concerned, this phenomenon is not detrimental to wine quality. 

The greatest impact that citrate fermentation has on wine, however, is linked to the production of diacetyl, which is responsible for a buttery aroma. Wines that undergo malolactic fermentation generally have a greater concentration of diacetyl than those that do not [148]. Moreover, this transformation is promoted by the prolonged contact with bacterial biomass or yeast lees. While moderate levels of diacetyl have a positive effect on aroma, high levels cause an unpleasant aroma, leading to spoilage [149]. Consequently, winemakers try to control diacetyl concentrations to enhance aroma by eliminating the microorganisms earlier in the process or, in contrast, they attenuate its impact by maintaining the wine with yeast lees. The final concentration of diacetyl in wine also depends on various factors, including bacterial strain, wine type, and sulphur dioxide and oxygen concentrations [149].

Analyses of the *O. oeni* genome showed the presence of the typical *cit* gene group, which includes genes that encode citrate lyase (*cit-DEF*), citrate lyase ligase (*citC*), oxaloacetate decarboxylase (*mae*) and the citrate transporter (*maeP* o *citP*) [150]. The genome also contains genes involved in the butanediol pathway (*ilvB, alsD, butA*).

## 5. *Brettanomyces*/*Dekkera bruxellensis* and Volatile Phenols 

**Figure 8:**
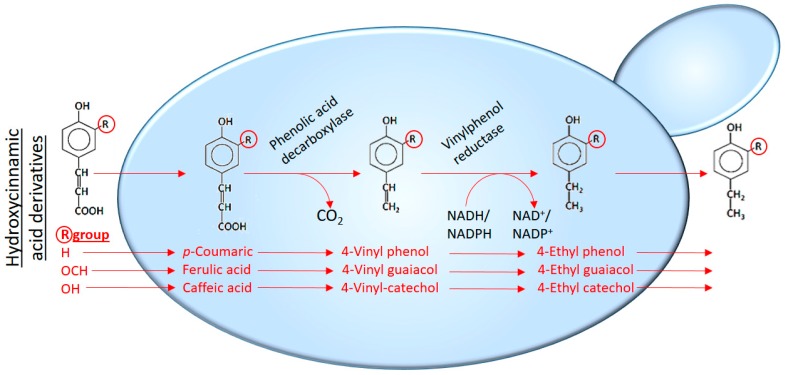
Formation of 4-ethyl derivatives from their correspondent hydroxycinnamic precursors by strains of the *Brettanomyces/Dekkera* genus.

*Brettanomyces* (teleomorph, *Dekkera*), with *B. bruxellensis* as the most frequently encountered representative, is considered a wine spoilage yeast due to the production of 4-ethylphenol and 4-ethylguaiacol, the most abundant off-aromas produced by this species, among others (4-ethylcatechol, 4-vinylguaiacol, 4-vinylphenol and 4-vinylcatechol). These phenolic off-flavours are described as horse sweat, humid leather, smoky, plastic, phenolic, medical, band-aid and poultry yard [151,152]. However, an increasing number of authors has reported that in particular cases, these yeasts can produce desired aromas that increase the flavour complexity of fermented beverages [153]. As stated before, 4-ethylphenol and 4-ethylguaiacol are the main phenolic off-flavours produced by *Brettanomyces.* They are produced in a two-step pathway (Figure 8) due to the decarboxylation of the corresponding hydroxycinnamic acids (caffeic, *p*-coumaric and ferulic acids), followed by a reduction of the intermediate 4-vinylphenols [154,155,156]. Hydroxycinnamic acids are widespread in plants, where they are found in leaves, fruits, seeds and roots [157]. In grapes, hydroxycinnamic acids primarily consist of caffeic, ferulic and *p*-coumaric acids [157] and are present at concentrations and proportions that depend on numerous viticultural factors such as grape variety, ripeness, sun exposure, orography, harvest date and geographical region, among others [158].

As *S. cerevisiae*, *B. bruxellensis* shares rather uncommon traits rarely combined in one species, such as high resistance to osmotic stress, ethanol, Crabtree effect, low pH and growth in oxygen-limited environments, that enable these peculiar yeasts to spread in alcoholic fermentations. In addition, *B. bruxellensis* is also able to hydrolyse and ferment complex sugars such as cellobiose and dextrins of (ligno)cellulose and wood, [159,160] which might help to explain how *B. bruxellensis* can survive for years in wooden casks used in wine ageing [161]. This also requires a β-glucosidase, which is also found in several *B. bruxellensis* strains [160,162]. *Brettanomyces* is mostly associated with barrel-aged red wines, but has also been found in Chardonnay and Sauvignon blanc, and sparkling wines. Concerning the most probable way for winery contamination with *Brettanomyces*, it is thought that ethyl phenols produced by *Brettanomyces* can serve as an attractant for insects and can be introduced to a winery by insect vectors such as *Drosophila melanogaster*, or by purchasing contaminated wine barrels [163].

Reported information concerning *B. bruxellensis* growth, physiology and off-flavours production seems to be contradictory, in part, due to the usage of different assay conditions, which are often far from real vinification conditions [164]. Phenolic off-flavours production has been related with growing *B. bruxellensis* cells [165,166,167], and, on the contrary, with the existence of viable but non-culturable cells which, in some cases, are able to produce them [168,169,170,171]. Recent studies [172] have reported that Pad1p (enzyme responsible for decarboxylating *p*-coumaric acid and converting it into 4-vinylphenol) decarboxylated *p*-coumaric acid during the early stages of growth and, subsequently, enzymes accumulated during the exponential growth phase (wherein the reduction in a second reaction step occurs) reduced of 4-vinylphenol to 4-ethylphenol.

Microorganisms that transform hydroxycinnamic acids are of relevance during alcoholic fermentation, where the resultant production of derivatives is often deleterious but can benefit other industries. For example, 4-vinylguaiacol is a very valuable product for use in a variety of industries and its bioconversion from ferulic acid by decarboxylating microorganisms is of potential interest [173].

## 6. Production of Off-Flavours by Lactic Acid Bacteria

LAB can also be responsible for the off-flavours in wine, including volatile phenols. Certain *Pediococcus* and *Lactobacillus* strains also have a role [174] in the production of these unpleasant compounds. De las Rivas et al. [175] analysed the capacity of LAB to produce volatile phenols in wine and described a PCR method for detecting bacteria with this potential. *L. plantarum, L. brevis*, and *P. pentosaceus* strains produced vinyl derivatives from hydroxycinnamic acids, but only *L. plantarum* strains produced the corresponding ethyl derivatives. *O. oeni, L. hilgardii*, and *Leu. mesenteroides* strains, in contrast, did not decarboxylate the hydroxycinnamic, *p*-coumaric and ferulic acids, suggesting that they are not responsible for the production of volatile phenols.

The production of undesirable aromas and flavours in wine described as “mousy” or “acetamide” has been associated with several LAB [176]. A mousy odour or flavour is specifically attributed to the production of three volatile heterocyclic compounds: 2-ethyltetrahydropyridine, 2-acetyltetrahydopyridine, and 2-acetylpyrroline. Certain winemaking conditions such as high pH (>3.5) or low sulphur dioxide levels can favour the growth of the bacterial strains involved in the production of these bases. This flaw has been associated with heterofermentative strains of *Lactobacillus*, in particular *L. hilgardii*, followed by *O. oeni* and *Pediococcus* strains, as well as some homofermentative *Lactobacillus* species. A mousy taint can render an unpalatable wine and cannot be eliminated. Very few studies have analysed the origin of this flaw and little is known about its repercussion on wine quality due to the complexity of the process, but also because it occurs in conjunction with other defects. For instance, the presence of d-fructose has been associated with the production of volatile heterocyclic compounds and it has been suggested that the formation of these compounds involves ornithine and lysine metabolism in the presence of ethanol, although much remains to be discovered regarding the mechanisms underlying this process [177].

## 7. Contribution of Oral Microbiota to Wine Aroma Perception 

The aroma perceived during wine intake not only depends on the wine microbial derived volatile composition; furthermore, once the wine is introduced in the oral cavity, a contact with several human physiological factors takes place, interfering in personal aroma perception. These physiological factors are the main responsible for the inter-individual differences observed during wine intake [178] and it includes respiratory flow, salivary flow and composition, oral cavity dimension, oral mucosa and oral microbiota. Between them, the influence and role of the oral microbiota is almost completely unknown. 

Oral microbiota is the second most complex community in the human body, after the colon [179], and it is formed by more than 1000 taxa [180]. Inside the oral cavity, several locations for microbial communities can be found, including the supragingival plaque (dental plaque), subgingival crevice (subgingival plaque), the tongue, cheeks (epithelial cells) and teeth [180]. The organisation of the bacterial community is based on the formation of aggregates called “biofilms”, made up by primary and secondary colonizers. The composition of these oral biofilm structures depends on several factors (i.e., pH, redox potential, salinity, etc.), determinant being the salivary flow, since saliva acts as the main nutrient source. However, food is also used by the oral microbiota as nourishment due to its great variety of nutrients. This, together with the idea of a progressive aroma compounds release due to an interaction of wine constituents with oral mucosa [181], suggests a plausible effect or oral microbiota enzymatic activity over glycosidic aroma precursors, resulting in the release of free volatile aroma compounds.

It is already known that several salivary enzymes are able to degrade some phenolic compounds [182]. Moreover, some in vitro studies with human saliva have demonstrated the role of salivary enzymes (β-glycosidases, esterases, etc.) in the degradation of free aroma volatiles [183,184,185]. However, to our knowledge, the effect of the oral microbiota enzymatic system at this level has not been studied and, to our knowledge, only one work has been focused on the study of the production of wine odorant aglycones from their precursors by oral microbiota enzymatic activity [186]. On one side, the ability of specific common oral bacteria to release aroma compounds from grape odourless glycosidic precursors by itself was demonstrated after incubation of the precursors (obtained from a white grape aroma precursor extract) with individual bacteria cultures. Concretely, it was observed that all oral bacteria employed in this study, including aerobic bacteria such as *Staphylococcus aureus*, anaerobe facultative bacteria such as *Streptococcus mutans*, and anaerobe bacteria such as *Fusobacterium nucleatum*, were able to release terpenes, benzenic derivatives and C6-alcohols in a bacteria species/strain and time-dependent manner (0–72 h) , as showed by subsequent head space-solid phase microextraction (HS-SPME) coupled to gas chromatography-mass spectrometry (GC-MS) analysis. On the other side, the effect of a real representative oral microbiota sample was also studied. To get this aim, Muñoz-González and colleagues [186] analysed the aroma release after incubation of aroma precursors with a pool of human saliva collected from different healthy donors. Incubations of precursor with sterile and non-enzymatic saliva were also carried out in order to discard other effects, and results demonstrated that the microbial fraction of human saliva was able to release aroma compounds from precursors, showing therefore a clear influence of this factor on wine aroma release during intake. However, the knowledge at this point it is still scarce and more studies are required. Additionally, inter individual differences on physiological factors, including oral microbiota composition, make difficult to establish a general trend on this field. 

In conclusion, aroma perception is an important factor driving wine quality and acceptance. The production of odorant molecules by oral microbiota enzymes from non-odorant precursors has been described for the first time, suggesting a significant impact on aroma generation and perception during wine consumption. However, the study [186] is preliminary and uses an in vitro approach, which has some limitations. Thus, more studies are suggested, in order to go deep into this unexplored field.

## 8. Future Perspectives and Conclusions

In this article, the role of microorganisms in winemaking, as determinant factors on wine aroma composition, has been reviewed. Considering winemaking as a dynamic process divided in two fermentation steps, the contribution of yeasts and bacteria with *S. cerevisiae* and *O. oeni* as reference species, respectively, has been described, but also the current knowledge about other wine-related non-conventional microbial species has been exposed. During the last decade, a massive number of works has been carried out to investigate the role and potential use of non-conventional yeasts and bacteria in winemaking [2,33]. These physiological studies have been crucial to better understand the complexity and potentiality of spontaneous and multistarter fermentations. Since several yeast and bacteria species have been studied at this level, now it is time to explore their genomic and transcriptional diversity, as the basis of their promising metabolic features [187,188,189]. As compared to genetic understanding of wine yeast strains, genetic advances in the LAB field is still taking its first steps. Considering the challenges usually encountered by winemakers to control malolactic fermentation, due to its contribution to wine sensorial properties, genetic improvement of malolactic starters might be of clear interest. Possible targets for improvement would be adaptation to harsh wine conditions or metabolic pathways involved in the production of sensory active compounds (e.g., diacetyl) [190]. 

In spite of the focus of this review, abridging the current knowledge about the incidence of microbial metabolism in wine aroma, our ostensible idea is to remark the importance of the holistic approaches to future studies in this field. Since its origin, oenology is a multidisciplinary science including chemical, microbiological and agronomical concepts, but it should also include human physiology to clarify the interaction of wine components (i.e. aroma compounds) with the human body. In addition, bioinformatics and network science are nowadays two of the main factors working to put this diverse information together, linking the microbial complexity of wine fermentation and humans with the chemical determinants of wine flavour; in other words, to connect metagenomics with metabolomics meaning [3]. In this context, this review also intends to advise that it appears important in the future to deeply understand the microbial contribution of oral bacteria for wine aroma conversion/release, and pointed out the need of more studies on this unknown research field.

Finally, a consortium of a dozen laboratories from five countries (USA, UK, Australia, China and Singapore) has embarked on a large collaborative project (Sc2.0) focused on the synthesis of the entire genome of a haploid laboratory yeast strain (S288c) of *S. cerevisiae* [191]. This international project is on track to produce the world’s first synthetic yeast by 2018. If successful, synthetic genomics will breathe new life into wine yeast researchers, wineries and consumers, among others.
